# Hot Tub-Associated Pseudomonas Folliculitis: A Case Report and Review of Host Risk Factors

**DOI:** 10.7759/cureus.10623

**Published:** 2020-09-23

**Authors:** Joanne S Jacob, Jaime Tschen

**Affiliations:** 1 Dermatology, Baylor College of Medicine, Houston, USA; 2 Dermatology, St. Joseph Dermatopathology, Houston, USA

**Keywords:** pseudomonas, folliculitis, hot, tub, pool, cutaneous, flora, diabetes

## Abstract

*Pseudomonas aeruginosa* folliculitis is an infection of the skin commonly associated with swimming pool and hot tub use. It often presents as outbreaks affecting multiple individuals using the same contaminated public water facility. We present a case report of a 50-year-old woman who developed pseudomonal folliculitis after using a hot tub with multiple family members. No other family member developed folliculitis. Factors contributing to susceptibility to *P. aeruginosa* infection are reviewed.

## Introduction

*Pseudomonas aeruginosa* folliculitis is an infection of the skin commonly associated with swimming pool and hot tub use. It is estimated that 67% of hot tubs and 63% of swimming pools are contaminated by *P. aeruginosa* at any single point [1]. Due to its association with public facilities, it often presents as outbreaks affecting multiple individuals [2]. We present a case report of a 50-year-old woman who developed pseudomonal folliculitis after using a hot tub with multiple family members. No other family member developed folliculitis. 

## Case presentation

A 50-year-old woman presented for evaluation of an itchy rash on the skin. Two days prior to presentation, the patient spent several hours in a hot tub that multiple family members also used on the same day. It appears the patient used the hot tub for a longer and more continuous time than her family members. No other family member developed a rash. The patient has an allergy to iodine and uses fexofenadine for seasonal allergies. She uses ibuprofen for occasional pain relief.

**Figure 1:**
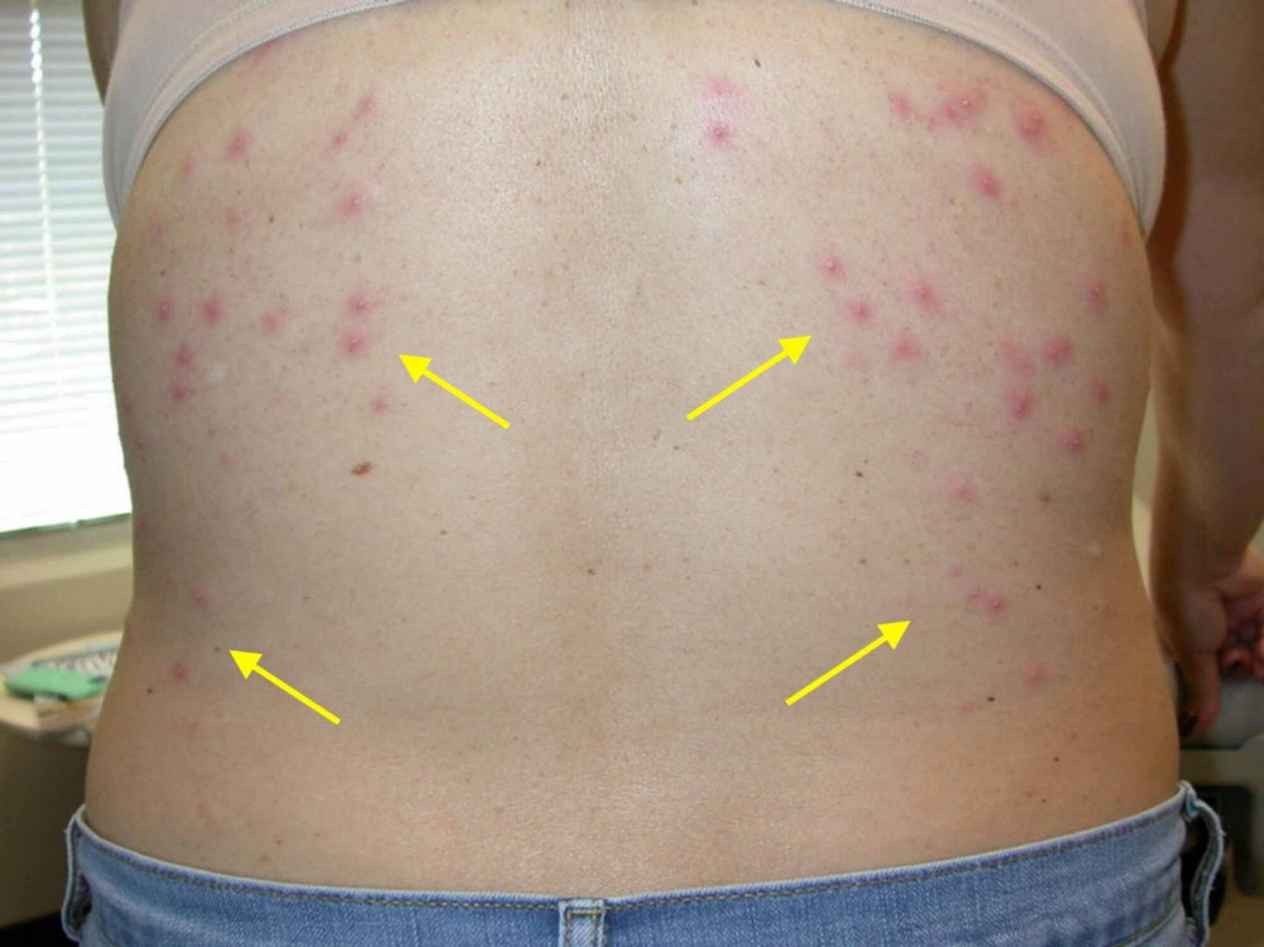
Clinical photo of patient's back Distribution of papules and pustules seen involves areas occluded by clothing or swim wear, which is characteristic of pseudomonal folliculitis.

**Figure 2:**
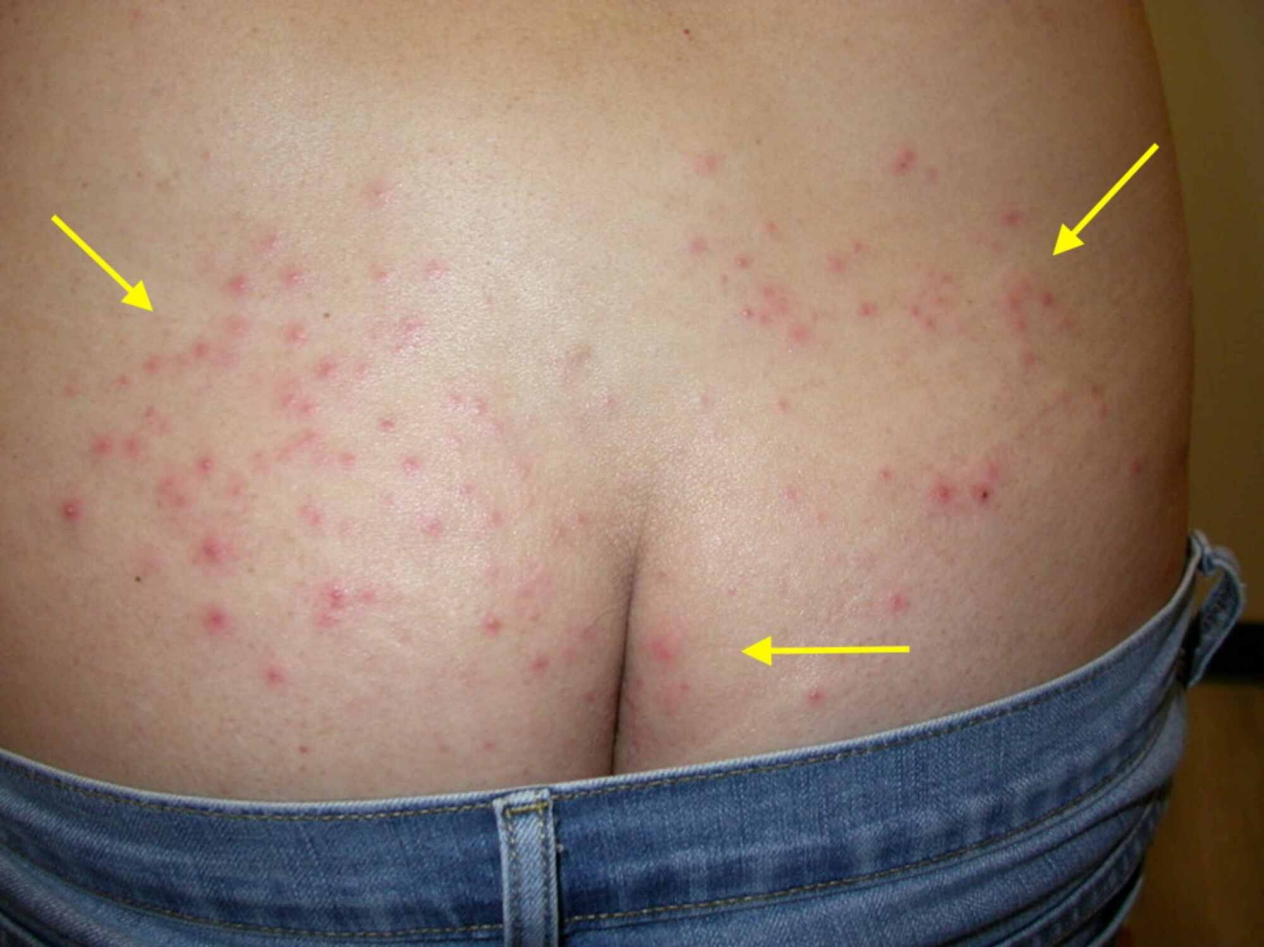
Clinical photo of patient's upper buttocks Consistent with the lesions on the back, distribution of pruritic papules and pustules is on clothing-occluded areas of the patient's skin.

**Figure 3:**
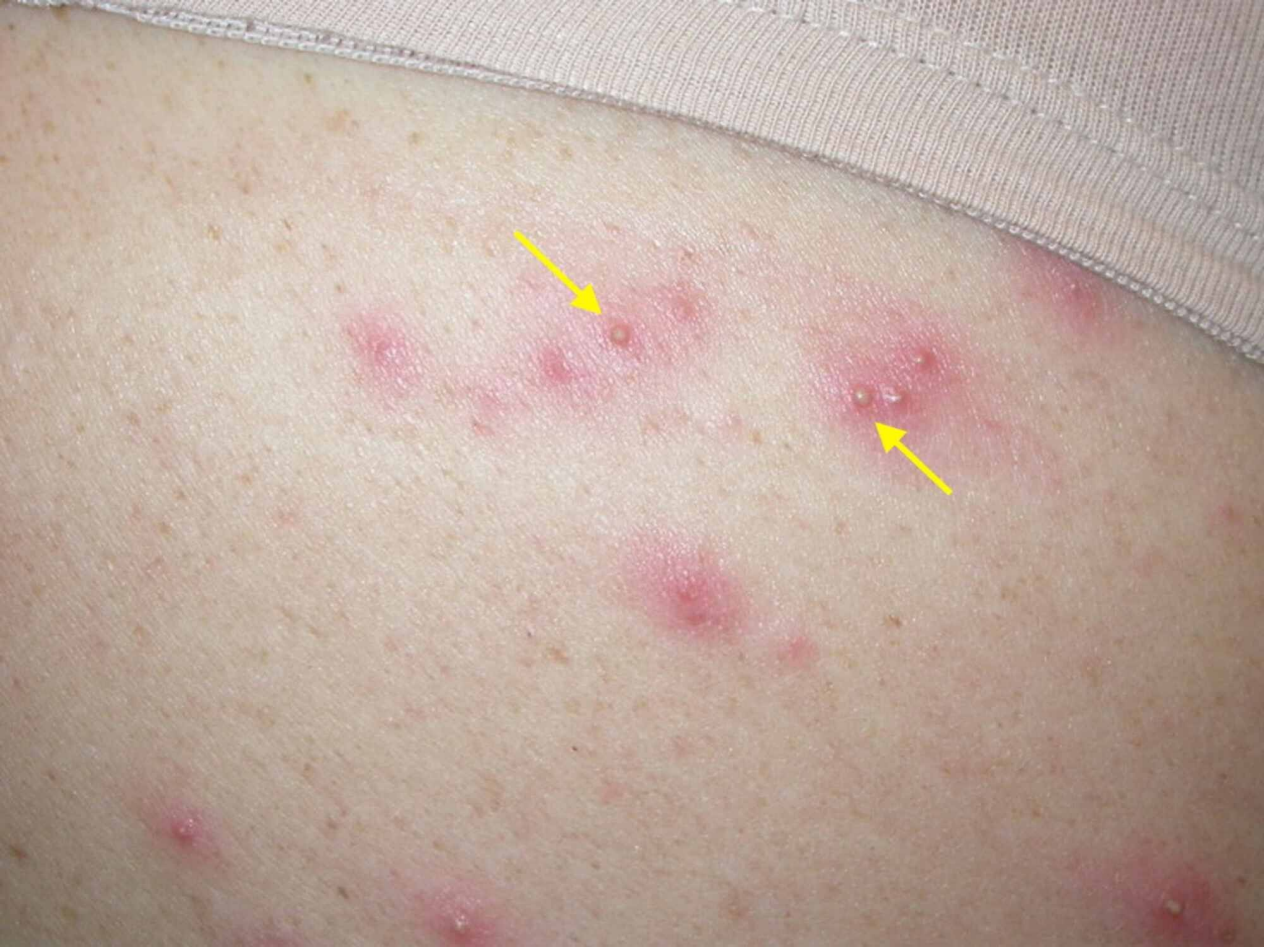
Magnified clinical image of lesions On closer examination, follicular arrangement of papules and pustules is more evident.

**Figure 4:**
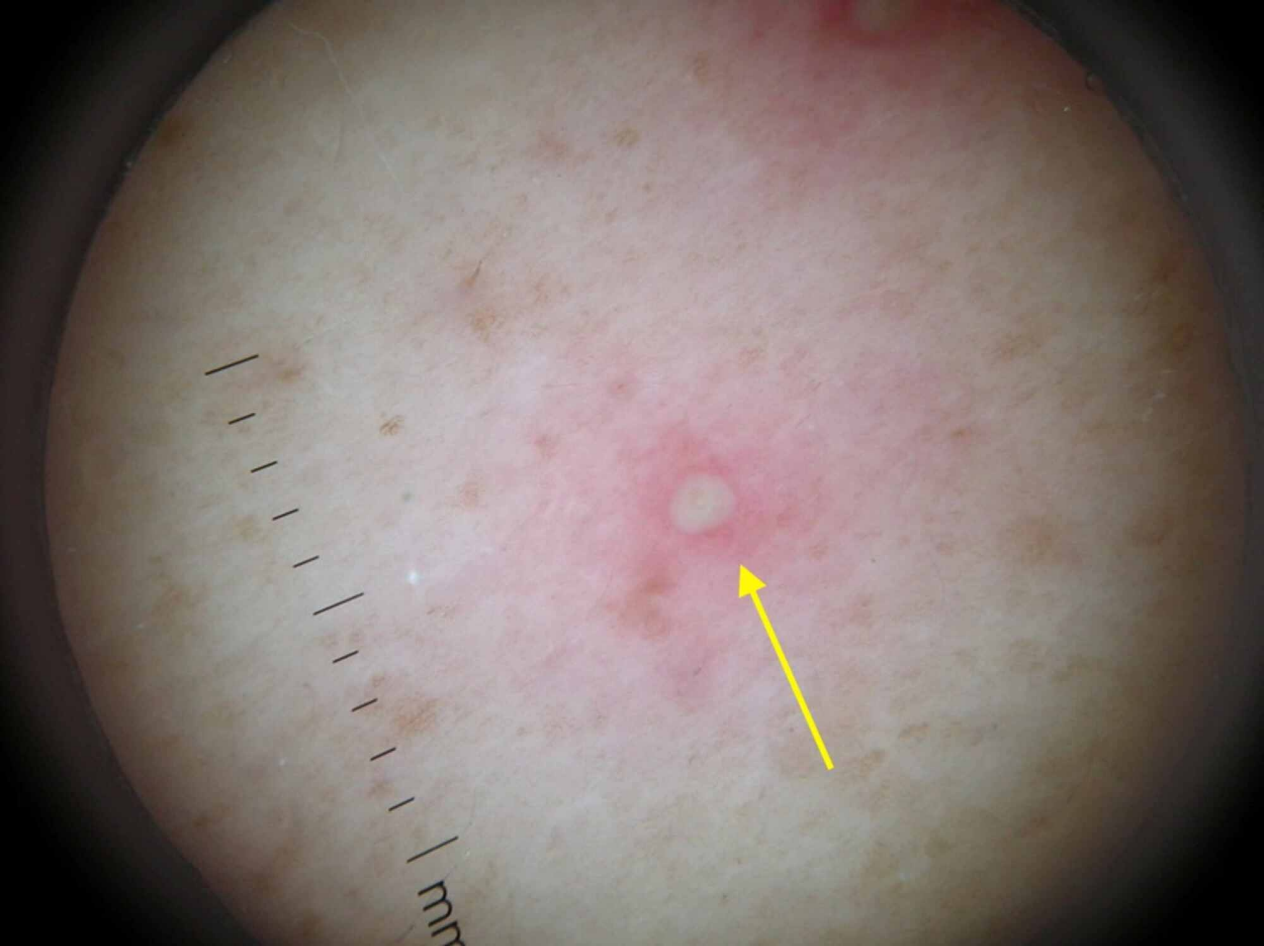
Dermoscopy of a lesion Dermoscopy of one lesion demonstrated a pustule with a central punctum on an erythematous base.

Physical examination revealed pruritic papules and pustules on the chest, back, and buttocks (Figures 1-3). Dermoscopy of a single lesion demonstrated a pustule with a central punctum on an erythematous base (Figure 4). Involved areas were occluded by swimwear when using the hot tub. A wound culture of a lesion on the back was collected. Clinical suspicion was for folliculitis, either by *Staphylococcus aureus* or *P. aeruginosa*. The patient was empirically prescribed oral combination therapy 160 mg trimethoprim and 800 mg sulfamethoxazole twice daily and topical neomycin/polymyxin B/bacitracin ointment twice daily.

The culture of the wound revealed *P. aeruginosa*. Antibiotic susceptibility showed the infectious organism to be broadly sensitive.

When the patient was called six days after presentation for follow-up of results and antibiotic susceptibility, the patient’s rash had resolved despite not being sensitive to trimethoprim-sulfamethoxazole combination therapy.

## Discussion

Pseudomonal folliculitis is an infection of follicular epithelium by *P. aeruginosa* that occurs with continuously wet and occluded skin. Thus, it is a common infection from hot tubs and swimming pools when the skin is occluded by swimwear. Within 48 hours of exposure, it presents as pruritic papules and papulopustules ranging from two to ten millimeters in diameter. It most often presents on the flanks, axillae, buttocks, and ears [2].

Management of pseudomonas folliculitis can vary with severity of infection. In most cases, lesions are self-limited and resolve without management in one to two weeks [2]. The infection was mostly likely self-limited in the presented patient, as the organism was not susceptible to the trimethoprim-sulfamethoxazole that was prescribed.

In cases in which the folliculitis does not self-resolve, a culture and antibiotic susceptibility testing can provide guidance on appropriate therapy. Antipseudomonal agents include antipseudomonal penicillins (ticarcillin and piperacillin), beta-lactamase inhibitor combinations (ticarcillin-clavulanate and piperacillin-tazobactam), carbapenems (doripenem, imipenem, and meropenem), cephalosporins (ceftazidime and cefepime), colistin, fluoroquinolones (ciprofloxacin or levofloxacin), and monobactams (aztreonam) [3].

Studies of *P. aeruginosa* found in public pools demonstrate considerable antibiotic resistance to agents such as aztreonam and imipenem [1]. The oral antibiotic group least associated with resistance and most commonly recommended is fluoroquinolones, specifically ciprofloxacin [2,4].

Pseudomonal folliculitis is known to affect large groups that use shared public water facilities like swimming pools and hot tubs. Factors influencing the prevalence of *P. aeruginosa* in a contaminated body of water can include biofilm formation on pool structures, seasonality, and water treatment processes [5]. Many factors can affect the risk for a certain individual to contract pseudomonal folliculitis. These factors include changes to cutaneous flora, length of exposure, skin trauma, and gender.

It is a recognized phenomenon that *P. aeruginosa* infections can occur with changes in the flora of the skin. Chronic medical conditions such as diabetes can deplete normal cutaneous flora. Oxidative stress from elevated blood glucose in diabetes leads to loss of protective cutaneous bacteria. This facilitates skin colonization by abnormal bacteria such as *P. aeruginosa.* Incidence of pathogenic bacterial colonization increases proportionally to blood glucose elevation [6].

Patients with impaired immune systems, either by malignancy or immunosuppressive medications, can also present with an imbalance of normal bacteria on the skin. One study demonstrated that when compared to a control group, patients with leukemia were predisposed to rapid colonization of gram-negative bacteria at all sampled body sites. This was thought to be attributable to a reduction in gram-positive bacteria that normally exclude pathogenic gram-negative bacteria, though the mechanism was not fully understood [7]. A similar phenomenon was seen in chronic antibiotic use, particularly tetracyclines [8]. These disruptions in protective cutaneous flora can leave an individual more vulnerable to water-borne exposure to *P. aeruginosa*.

Further associations have been made between pseudomonal folliculitis and behaviors in pools or hot tubs. Of these, the length of time in the water has the greatest association with skin infection [5,9]. It is hypothesized that water absorption of the stratum corneum increases proportionally with time submerged in water. Bacterial invasion into the skin is facilitated by the enhanced permeability of the stratum corneum after immersion in water [9]. 

Physical trauma to the skin also contributes to infection by *P. aeruginosa*. Physical or thermal burns to the skin disrupt the stratum corneum and impair the skin’s defense to bacterial infection [2]. Sunburns from ultraviolet radiation have a different pattern of involvement and can affect all levels of the epidermis. When associated with sloughing, it can also lead to increased risk of infection; this is a different mechanism from thermal burns [10].

Differences in gender of the individual can also influence the risk of pseudomonas folliculitis. In studies of pseudomonal folliculitis outbreaks, there were more women affected than men [5,9]. Hypotheses to explain this disparity include intrinsic differences in skin flora between men and women and differing topical product use, such as lotions and deodorants [9]. Studies have demonstrated both qualitative and quantitative differences in the cutaneous flora between men and women when controlling for differences in lotion and deodorant use [11]. A combination of both factors may contribute to the greater incidence of pseudomonal folliculitis in females.

In the presented patient, female gender, differences in skin flora, and length of time of exposure are three possible theories for why she alone developed *P. aeruginosa* folliculitis. Her family members included both males and females so, while gender may have still contributed, it would not be the sole differentiating factor.

Though this patient is not known to have diabetes or to be immunocompromised, her cutaneous flora may differ from her family members. This unknown intrinsic difference could have contributed to her risk of bacterial carriage.

While it was difficult to quantify total time of exposure of different family members, it appears that she used the hot tub for a greater amount of continuous submersion and total time of use. This would have allowed for greater permeability of the stratum corneum and facilitated development of pseudomonas folliculitis.

## Conclusions

Numerous factors may influence an individual’s risk of developing *P. aeruginosa* folliculitis from an environmental exposure. These risk factors include changes to cutaneous flora, female gender, length of exposure, and skin trauma. It would be impractical to recommend all women avoid extended hot tub use. Additionally, patients with unhealed thermal burns of skin are not likely to use hot tubs or public pools. Thus, counseling should be directed to those with diabetes, immunocompromised states, or trauma of the skin. These populations can be educated on the risk of infection with extended submersion in public pools or hot tubs.
